# Sensory Reactivity Symptoms Are a Core Feature of ADNP Syndrome Irrespective of Autism Diagnosis

**DOI:** 10.3390/genes12030351

**Published:** 2021-02-27

**Authors:** Paige M. Siper, Christina Layton, Tess Levy, Stacey Lurie, Nurit Benrey, Jessica Zweifach, Mikaela Rowe, Lara Tang, Sylvia Guillory, Danielle Halpern, Ivy Giserman-Kiss, Maria Del Pilar Trelles, Jennifer H. Foss-Feig, Silvia De Rubeis, Teresa Tavassoli, Joseph D. Buxbaum, Alexander Kolevzon

**Affiliations:** 1 Seaver Autism Center for Research and Treatment, Icahn School of Medicine at Mount Sinai, New York, NY 10029, USA; christina.layton@mssm.edu (C.L.); tess.levy@mssm.edu (T.L.); staceymlurie@gmail.com (S.L.); nurit.benrey@mssm.edu (N.B.); jessica.zweifach@mssm.edu (J.Z.); sylvia.guillory@mssm.edu (S.G.); danielle.halpern@mssm.edu (D.H.); pilar.trelles@mssm.edu (M.D.P.T.); jennifer.foss-feig@mssm.edu (J.H.F.-F.); silvia.derubeis@mssm.edu (S.D.R.); joseph.buxbaum@mssm.edu (J.D.B.); alexander.kolevzon@mssm.edu (A.K.); 2 Department of Psychiatry, Icahn School of Medicine at Mount Sinai, New York, NY 10029, USA; 3 Mindich Child Health and Development Institute, Icahn School of Medicine at Mount Sinai, New York, NY 10029, USA; 4 Ferkauf Graduate School of Psychology, Yeshiva University, Bronx, NY 10461, USA; 5 Radiology and Biomedical Imaging, University of California San Francisco, San Francisco, CA 94143, USA; rowem513@gmail.com; 6 David Geffen School of Medicine at UCLA, Los Angeles, CA 90095, USA; lara.cm.tang@gmail.com; 7 Neurodevelopmental and Behavioral Phenotyping Service, National Institutes of Mental Health, Bethesda, MD 20814, USA; ivygise@gmail.com; 8 Friedman Brain Institute, Icahn School of Medicine at Mount Sinai, New York, NY 10029, USA; 9 School of Psychology and Clinical Language Sciences, University of Reading, Berkshire RG6 6BZ, UK; teresa.tavassoli@gmail.com; 10 Department of Genetics and Genomic Sciences, Icahn School of Medicine at Mount Sinai, New York, NY 10029, USA; 11 Department of Neuroscience, Icahn School of Medicine at Mount Sinai, New York, NY 10029, USA; 12 Department of Pediatrics, Icahn School of Medicine at Mount Sinai, New York, NY 10029, USA

**Keywords:** ADNP syndrome, sensory reactivity, autism spectrum disorder

## Abstract

*Background*: Activity dependent neuroprotective protein (ADNP) syndrome is one of the most common single-gene causes of autism spectrum disorder (ASD) and intellectual disability, however, the phenotypes remain poorly described. Here we examine the sensory reactivity phenotype in children and adolescents with ADNP syndrome. *Methods:* Twenty-two individuals with ADNP syndrome received comprehensive clinical evaluations including standardized observations, caregiver interviews, and questionnaires to assess sensory reactivity symptoms. Relationships between sensory symptoms and age, sex, ASD, IQ, and adaptive behavior were examined. Genotype-phenotype correlations with the recurrent p.Tyr719* variant were also explored. *Results:* Sensory reactivity symptoms were observed and reported in all participants. A syndrome-specific phenotype was identified, characterized by high levels of sensory seeking across tactile, auditory, and visual domains. Tactile hyporeactivity, characterized by pain insensitivity, was reported in the majority of participants. Sensory symptoms were identified across individuals regardless of age, sex, IQ, adaptive ability, genetic variant, and most importantly, ASD status. No significant differences were identified between participants with and without the recurrent p.Tyr719* variant on any sensory measure. *Conclusions:* Sensory reactivity symptoms are a common clinical feature of ADNP syndrome. Quantifying sensory reactivity using existing standardized measures will enhance understanding of sensory reactivity in individuals with ADNP syndrome and will aid in clinical care. The sensory domain may also represent a promising target for treatment in clinical trials.

## 1. Introduction

Activity dependent neuroprotective protein *(ADNP*) syndrome (OMIM: 615873) is an autosomal dominant neurodevelopmental disorder characterized by mild-to-severe intellectual disability (ID), autism spectrum disorder (ASD), speech and motor delays, and a variety of medical comorbidities [1,2,3]. The *ADNP* gene codes for activity dependent neuroprotective protein, a ubiquitously expressed protein involved in chromatin remodeling [4,5] and synaptic function [6,7,8]. *ADNP* is one of many genes involved in chromatin remodeling that has been linked to neurodevelopmental disorders [9].

ASD is present in one half to two thirds of individuals with ADNP syndrome [1,3] and ADNP syndrome accounts for approximately 0.2% of all cases of ASD [2]. The ASD phenotype in ADNP syndrome is characterized by less social impairment and more frequent stereotyped motor behaviors when compared to individuals with idiopathic ASD or those with other genetic syndromes associated with ASD [1]. Social deficits in ADNP syndrome were associated with verbal impairment and therefore memory and learning deficits were described as a prominent feature of the syndrome. This is consistent with significantly higher rates of intellectual disability in individuals with ADNP syndrome relative to rates observed in idiopathic ASD. Similar to other monogenic causes of ASD, ADNP syndrome is associated with various medical (e.g., gastrointestinal problems, hypotonia), behavioral (e.g., externalizing symptoms), and psychiatric (e.g., obsessive compulsive behavior, mood disorders) comorbidities [3]. One distinguishing symptom appears to be early tooth eruption, which is present in ~80% of individuals with ADNP syndrome [10].

In light of a growing body of research describing specific sensory phenotypes in neurodevelopmental syndromes [11,12,13,14], this study provides an in-depth prospective examination of the sensory phenotype in children and adolescents with ADNP syndrome and the relationship between sensory phenotypes and both clinical and demographic factors. The Diagnostic and Statistical Manual of Mental Disorders, 5th, Edition (DSM-5) [15] criteria for ASD broadly defines sensory reactivity symptoms within the Restricted, Repetitive Behavior (RRB) domain (“Hyper- or hyporeactivity to sensory input or unusual interests in sensory aspects of the environment”). Two of four RRBs and all three social communication criteria are required for a diagnosis of ASD. Literature suggests sensory symptoms are among the earliest clinically observable predictors of a neurodevelopmental disorder [16,17] and are present in up to 90% of individuals with ASD [18,19]. Sensory symptoms are also correlated with lower levels of adaptive functioning [20] and higher levels of anxiety [21,22] and attention problems [23,24], all of which are commonly observed in individuals with ADNP syndrome. Improved identification and awareness of sensory symptoms in individuals with ADNP syndrome may offer earlier intervention and improved quality of life for individuals with ADNP syndrome and their families. In addition, understanding the sensory phenotype within ADNP syndrome can inform the development of personalized treatment approaches.

## 2. Materials and Methods

### 2.1. Participants

Twenty-two individuals (10 female) between the ages of 2 and 17 years old (Mean age = 7.72, SD = 4.04) participated in comprehensive clinical evaluations as part of an ADNP syndrome natural history study. Participants all have likely pathogenic or pathogenic variants detected by next generation sequencing performed at Clinical Laboratory Improvement Amendments (CLIA) certified laboratories. Variants were annotated according to the Human Genome Variation Society Guidelines (HGVS) and mapped to the RefSeq transcript NM_015339.4 (Table S1). The American College of Medical Genetics and Genomics and Association for Molecular Pathology (ACMG-AMP) Guidelines [25] were used to interpret each variant. Vision and hearing problems were reviewed to ensure participants did not have interfering uncorrected sensory impairment (Table S2).

### 2.2. Ethics Declaration

The study was approved by the Mount Sinai Program for the Protection of Human Subjects (Study: 98-0436, Assessment Core for phenotyping approved annually since 1998). Parents or legal guardians of all participants signed written informed consent for participation. Assent was obtained where applicable. 

### 2.3. Clinical Evaluation

Comprehensive clinical evaluations were completed using a battery of well-validated instruments commonly used in the assessment of individuals with ASD, ID, and related conditions [26]. All participants received a psychiatric evaluation by a board-certified child and adolescent psychiatrist and gold-standard autism diagnostic testing by clinical psychologists with established research reliability. Using DSM-5 [15] diagnostic criteria for ID, cognitive and adaptive tests assessed the presence and severity of ID. Cognitive tests included the Mullen Scales of Early Learning [27] or the Stanford-Binet Intelligence Scales, 5th Edition [28]. The Vineland Adaptive Behavior Scales, 3rd Edition [29] was used as a measure of adaptive behavior. DSM-5 diagnosis for ASD was determined based on a consensus diagnosis from the psychiatric evaluation, and gold standard diagnostic assessments, including the Autism Diagnostic Observation Schedule, 2nd Edition (ADOS-2) [30] and the Autism Diagnostic Interview-Revised (ADI-R) [31]. The ADOS-2 is a semi-structured 45–60-min direct assessment of an individual’s communication, socialization, and restricted/repetitive behaviors. The ADI-R is a comprehensive diagnostic interview conducted with a caregiver to assess current and past symptoms in the following domains: socialization, communication, restricted and repetitive behavior, and age of onset. Both the ADOS-2 and ADI-R offer clinical cutoff scores for a classification of ASD based on extensive research in individuals with ASD relative to individuals with other developmental delays [32,33]. Both the ADOS-2 and ADI-R were administered by research reliable clinicians.

Sensory reactivity was measured using standardized observations, caregiver interviews, and questionnaires, including:

*Sensory Assessment for Neurodevelopmental Disorders (SAND)* [34]. A clinician-administered observation and corresponding caregiver interview that quantifies sensory hyperreactivity, hyporeactivity, and seeking across visual, tactile, and auditory modalities.

Higher scores indicate a greater number of symptoms. Normed cut-off scores are rated as within normal limits, elevated (+1 SD), or clinically significant (+2 SDs). Z-scores were also examined and were based on normative data from over 300 individuals, including typically developing (TD) controls and individuals with ASD without known genetic etiology [i.e., idiopathic ASD (iASD)]. The SAND produces an overall Total Score based on a composite of all Observation and Interview codes as well as composite scores for six scales (Hyperreactivity, Hyporeactivity, and Seeking Domains; Visual, Tactile, and Auditory Modalities), and nine subscales (e.g., Visual Hyperreactivity, Visual Hyporeactivity, Visual Seeking, etc.).

*Short Sensory Profile (SSP)* [35]. A caregiver questionnaire that assesses sensory processing in everyday settings. Lower scores indicate a greater number of symptoms. Normed cut-off scores indicate typical performance, probable sensory differences (-1SD) definite differences (-2SDs) across seven scales.

*Autism Diagnostic Observation Schedule, 2nd Edition (ADOS-2)* [30]. Scores on the “unusual sensory interests in play material/person” item were examined. 

*Autism Diagnostic Interview-Revised (ADI-R)* [31]. Scores on items relevant to sensory processing were examined: (i) “unusual sensory interests,” (ii) “abnormal, idiosyncratic, negative response to specific sensory stimuli,” and (iii) “undue general sensitivity to noise.” Algorithms include responses to both current and historical (“ever”) behavior. Item-level scoring is the same as described for the ADOS-2.

### 2.4. Analysis

A variety of statistical analyses were used to examine the sensory phenotype within individuals with ADNP syndrome and to assess whether sensory symptoms were a characteristic of the syndrome broadly or associated features such as ASD, ID, adaptive ability or genetic variant. First, percentages were calculated to quantify the frequency of sensory symptoms overall and by individual measures. Then, group differences were examined in the individuals with ADNP syndrome who received a diagnosis of ASD (*n* = 11) compared to those who did not (*n* = 11). To do this, multivariate analysis of variance (MANOVAs) were run between groups on SAND, SSP, and ADI-R scores. In addition, to assess if the severity of ASD was associated with sensory symptoms, Pearson’s correlation coefficients (*r*) were calculated between ADOS-2 comparison scores and sensory reactivity symptoms. Spearman’s rank-order (rs) correlations assessed the relationship between sensory symptoms and ordinal variables from the ADOS-2 and ADI-R. A one sample *t*-test was used for the single ADOS-2 sensory item. Further exploratory analyses examined if other comorbidities (e.g., ID, adaptive functioning) or demographic characteristics were associated with sensory symptoms. To do so, Pearson’s correlation coefficients were calculated between IQ/DQ, Vineland-3 scores, and scores on sensory measures. Pearson’s correlation coefficients were also used to assess the relationship between age and sensory symptoms. Lastly, to assess whether sex was correlated with sensory symptoms a point-biserial correlation was completed. To minimize the likelihood of type II error, Bonferroni adjustments were not used [36,37].

## 3. Results

### 3.1. Genetic Testing

**Figure 1:**
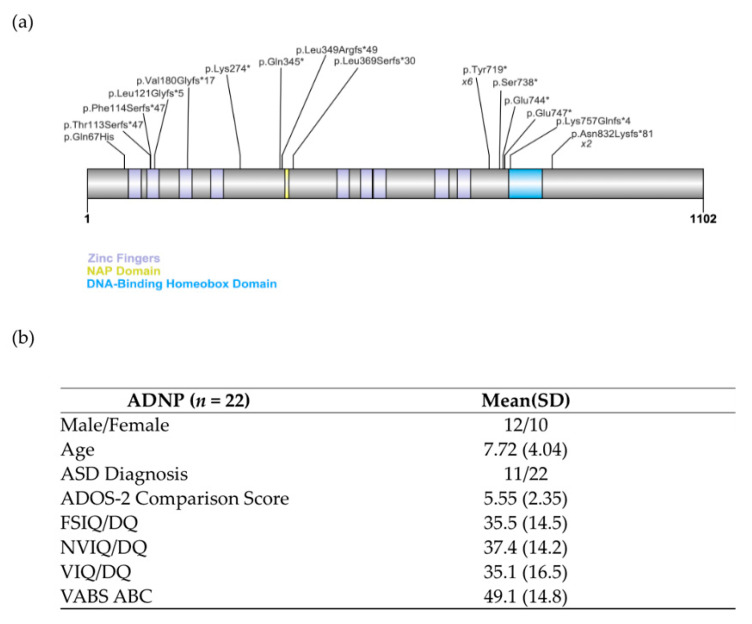
(**a**) ADNP variants in the cohort. The zinc fingers (purple), NAP domain (yellow), and DNA-Binding Homeobox domain (blue) are shown as reported in Uniprot Q9H2P0, (ADNP_Human). Two pathogenic variants are represented by more than one individual in the cohort, p.Tyr19* in six individuals, and p.Asn832Lysfs*81 in two. Not pictured: 5’UTR_EX2del. (**b**) Participant demographics. ADOS-2 comparison scores range from 1–10 with higher numbers reflecting greater symptom severity. IQ and Vineland scores are reflected as standard scores (*M* = 100; *SD* = 15). Developmental Quotients (DQs) were calculated by dividing age equivalents by chronological age for participants above the normed age range on the Mullen and unable to complete the Stanford-Binet.

Participants (*n* = 22) all carried variants classified as pathogenic or likely pathogenic. Variants include 10 nonsense, 10 frameshift and one missense variant, and one partial deletion (Figure 1a). Within the nonsense variants, 6 carry the recurrent p.Tyr719* variant and there were 2 individuals with the recurrent frameshift variant, p.Asn832Lysfs*81. The partial deletion encompasses the 5’ UTR through the second coding exon. The missense variant is located in coding exon 2 and functional studies done clinically through Ambry’s Translational Genomics Lab have shown that this alteration leads to in-frame skipping of coding exon 2 (c.109_201del, p.D37_Q67del). De novo status has been confirmed in 19 cases; three cases did not have de novo confirmation. The three variants without de novo status confirmed are classified as pathogenic and one is a recurrent variant.

### 3.2. Participant Characteristics

All participants met DSM-5 criteria for ID. Ten presented with severe-to-profound ID (IQ/DQ < 40), 10 with moderate ID (IQ/DQ 40–55) and 2 with mild ID (IQ/DQ 55–69). Standard scores on the Vineland Adaptive Behavior Composite [38,39] ranged from 26–68 (all < 2nd percentile). Half the sample (*n* = 11) met DSM-5 criteria for ASD based on a consensus diagnosis determined by psychiatric evaluation, ADOS-2 and ADI-R (Figure 1b).

### 3.3. Frequency and Type of Sensory Symptoms by Measure

#### 3.3.1. SAND

**Figure 2:**
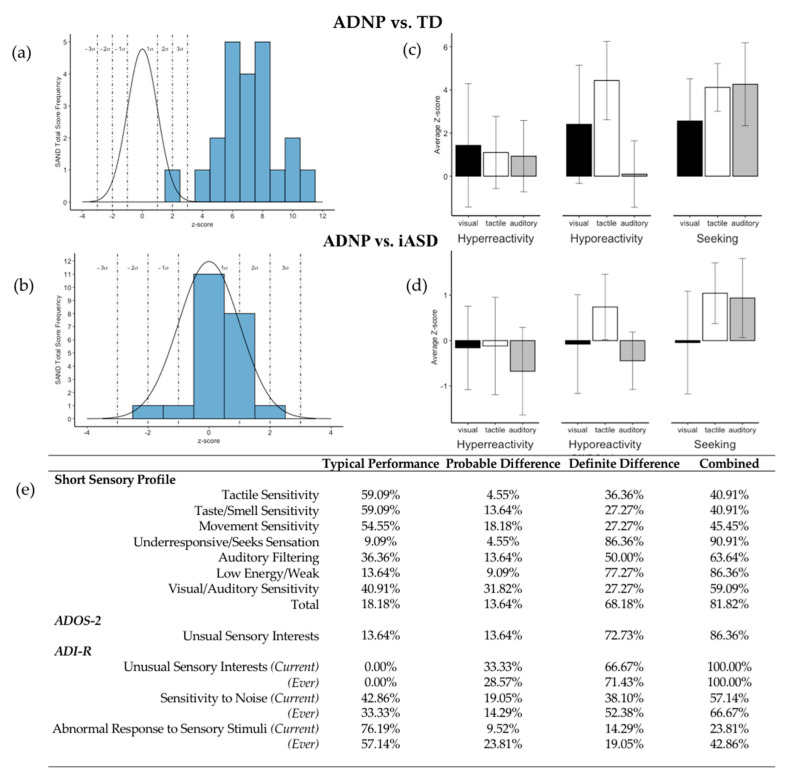
(**a**) Score distributions for individuals with ADNP syndrome relative to TD controls and (**b**) iASD from a normative sample. Dashed limit lines represent one, two, and three standard deviations above and below the mean. (**c**) Sensory hyperreactivity, hyporeactivity and seeking within visual, tactile, and auditory modalities based on TD z-scores. (**d**) Sensory hyperreactivity, hyporeactivity and seeking within visual, tactile, and auditory modalities based on iASD z-scores. Z-scores have a mean of 0 where +1 indicates 1 SD above the mean. (**e**) Frequency of sensory behaviors on the SSP, ADOS-2, and ADI-R. The ‘combined difference’ column reflects the sum of the ‘probable difference’ and ‘definite difference’ columns. Abbreviations: ADNP: Activity dependent neuroprotective protein; TD: typically developing; iASD: idiopathic autism spectrum disorder.

SAND total scores fell in the clinically significant range (+2SD) for 21/22 participants and in the elevated range (+1SD) for one participant. Sensory seeking fell within the clinically significant range for 96% of participants. Commonly observed seeking behaviors included mouthing objects, rubbing objects to skin, visual inspection, repetitive seeking of sounds (using objects and/or voice), and placing noisemaking objects near ears. Clinically significant levels of hyporeactivity were present in 11/22 (50%) participants and elevated in 2 additional participants. Scores in this domain were driven by the presence of pain insensitivity. On the SAND interview, 81% of parents reported a high pain/temperature threshold, which was observed in 62% of the sample during the SAND observation (Table 1). The observation directly assesses response to temperature using warm and cold packs. Clinically significant levels of overall hyperreactivity was present in 5/22 (23%) and elevated in 4 additional participants. Mean total scores were > 3 SDs higher than TD norms (Figure 2a) and similar to overall iASD sensory abnormalities (Figure 2b). Sensory seeking was > 2 SDs higher than the TD norms across visual, tactile, and auditory domains, and approximately 1 SD higher than iASD norms in tactile and auditory domains (Figure 2c,d). Tactile hyporeactivity (e.g., high pain/temperature threshold), was approximately 4 SDs higher than the TD norms and 1 SD higher than the iASD norms. Similar profiles were observed between ADNP and iASD norms in visual and tactile hyperreactivity, visual hyporeactivity, and visual seeking domains (Figure 2c,d).

#### 3.3.2. SSP

Probable to definite sensory differences were reported in 82% of the sample on the SSP, with underresponsiveness/seeks sensation (91%) and low energy/weak (87%) symptoms reported most frequently. Auditory filtering (e.g., appears not to hear when spoken to; poor response to name) and visual/auditory sensitivity were reported in over half the sample (Figure 2e). Tactile sensitivity, taste/smell sensitivity, and movement sensitivity were endorsed in less than half the sample.

#### 3.3.3. ADOS-2

Scores of 0 indicate no unusual sensory interests/behaviors, 1 indicates several possible sensory interests and/or one clear occurrence (Figure 2e, “probable difference”), 2 indicates definite sensory interests with at least two occurrences, and 3 reflects definite sensory interests that may have interfered with the assessment. Scores of 2 and 3 are combined into the “definite difference” category in Figure 2e. A minimum of one clear sensory seeking behavior was observed in 86% of participants, and at least two sensory seeking behaviors were observed in 73% of participants during the observation.

#### 3.3.4. ADI-R

All parents reported both current and historical sensory seeking behavior. Severe sensory seeking (code of 2 or 3) was reported as a current problem in 67% of the sample (Figure 2e) and as a historical problem by 76%. Responses to sensory hyperreactivity items indicated 71% of the sample had a history of noise sensitivity that persisted currently in 62% and causes significant distress in 38%. A quarter (24%) of participants currently display abnormal responses to specific sensory stimuli and 43% by history.

In 20 of 22 participants (91%), sensory reactivity abnormalities were identified on every measure. The remaining 2 participants displayed clinically significant symptoms on 3 of 4 measures.

### 3.4. Group Differences and Correlations with Clinical, Demographic, and Genetic Factors

#### 3.4.1. ASD

Group comparisons revealed that ASD diagnosis was not associated with differences in SAND or SSP scores. On the ADI-R, the unusual sensory interests and sensitivity to noise items (coded as currently shows behavior and ever showed behavior) showed no differences between individuals with ADNP syndrome with and without ASD. However, individuals with ASD were more likely to have abnormal responses to specific stimuli currently (*p* = 0.027; *n* = 5/10, versus none without ASD); the difference did not reach significance for the “ever” behavior code (*p* = 0.071). Additionally, ASD diagnosis did not show significant differences on the ADOS-2 sensory item (*p* = 0.641). Pearson’s correlations revealed that ASD severity (ADOS-2 comparison score) was not significantly correlated with any sensory measure.

#### 3.4.2. Cognitive and Adaptive Functioning

IQ/DQ scores were not correlated with SAND or ADOS-2 sensory scores. IQ/DQ was correlated with SSP total score (*r* = 0.441, *p* = 0.040), SSP auditory/visual sensitivity (*r* = 0.504, *p* = 0.017), and underresponsive/seeks sensation (*r* = 0.677, *p* = 0.001) scores. Q/DQ was significantly correlated with ADI-R current scores on the unusual sensory interests’ item (*r* = 0.510, *p* = 0.018). Overall adaptive behavior was significantly correlated with SSP total score (*r* = 0.584, *p* = 0.004), auditory/visual sensitivity (*r* = 0.625, *p* = 0.002), underresponsive/seeks sensation (*r* = 0.545, *p* = 0.009), and tactile sensitivity (*r* = 0.580, *p* = 0.005) scores. Adaptive behavior was not correlated with SAND, ADOS-2, or ADI-R scores.

#### 3.4.3. Age and Sex

**Figure 3:**
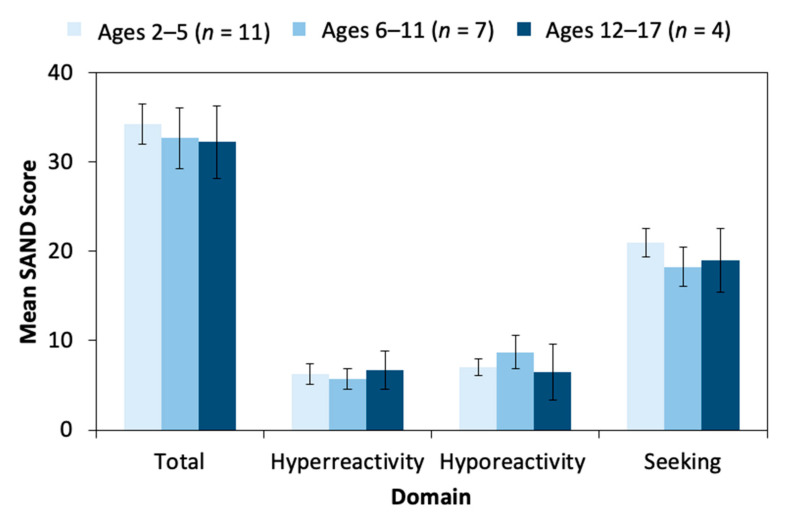
Sensory symptoms within three age cohorts reflecting early childhood, middle childhood, and adolescence. Results display stability in SAND scores across age groups.

There was no correlation between age and SAND (Figure 3) or ADOS-2 scores. Age was significantly correlated with SSP total score (*r* = −0.593, *p* = 0.004), tactile sensitivity (*r* = −0.568, *p* = 0.006), auditory/visual sensitivity (*r* = −0.637, *p* = 0.001), and movement sensitivity (*r* = −0.442, *p* = 0.039), with older individuals having more sensory symptoms. Age was significantly correlated with ADI-R abnormal responses to specific sensory stimuli current (*r* = 0.597, *p* = 0.004) and ever (*r* = 0.515, *p* = 0.019) scores. Sex was not correlated with scores on any sensory measure.

#### 3.4.4. Genotype-Phenotype

There were no significant differences between participants with and without the recurrent p.Tyr719* variant on any sensory measure.

## 4. Discussion

Here we describe a comprehensive prospective characterization of the sensory phenotype in 22 individuals with ADNP syndrome. Consistent with previous studies, all participants presented with mild-to-profound ID and half met DSM-5 criteria for ASD. A distinct phenotype was identified characterized by high levels of sensory seeking across tactile, auditory, and visual domains. High levels of seeking differentiate sensory features in ADNP syndrome from other syndromes associated with ASD. For example, Phelan-McDermid syndrome has been characterized by high levels of hyporeactivity and low levels of hyperreactivity [13,14]. Tactile hyporeactivity also was reported in the majority of cases and driven by pain insensitivity, at relatively higher rates than previously reported in the syndrome [3]. Pain insensitivity has been described in several other genetic causes of ASD including Phelan-McDermid syndrome [13], FOXP1 syndrome [11], Prader Willi syndrome [40], Dup15q syndrome [41] and Rett Syndrome [42]. Recognizing pain insensitivity as a common feature in individuals with genetic syndromes is important given safety concerns associated with high pain thresholds, particularly in individuals with language impairment and ID. Interestingly, pain insensitivity was common in this cohort, despite low levels of hyporeactivity in auditory and visual modalities. Our findings did not replicate previous literature describing a more severe phenotype associated with the recurrent p.Tyr719* variant [3]; however, results are consistent with Breen et al. 2020 [43], which included some participants in this cohort and showed no phenotypic differences based on methylation group.

ASD diagnosis and severity did not impact sensory symptoms identified by the SAND, SSP, or ADOS-2, indicating that the sensory phenotype in ADNP is generalizable across the syndrome, rather than driven by a subset with ASD. On the ADI-R, one difference was identified in abnormal response to specific sensory stimuli. This item probes distress in response to a particular, predictable stimulus, thus encompassing repetitive and restricted interests more broadly. Cognitive functioning, adaptive behavior, age, and sex did not impact SAND or ADOS-2 scores, both of which capture direct observation of symptoms. Interestingly, results from the SAND suggest a preservation of sensory symptoms with age, which is consistent with recent studies in individuals with idiopathic ASD demonstrating stability of sensory symptoms throughout early and middle childhood [44,45,46]. IQ/DQ, adaptive behavior, and age were correlated with several SSP scales, suggesting that the higher the cognitive or adaptive level and older the individual, the fewer abnormal sensory responses parents reported. In contrast, on the ADI-R unusual sensory interests item, results suggested higher cognitive and adaptive ability was related to a greater number of reported sensory interests. Differences in reported sensory behaviors and associations are likely measure dependent. For example, the SSP includes questions related to common comorbidities such as hypotonia (e.g., low energy/weak) and ADHD (e.g., jumps from one activity to another), which may impact sensory processing, but likely do not reflect primary sensory symptoms. Further, a comparison of current versus historical codes on the ADI-R suggests that certain sensory symptoms may improve over time, particularly when ASD is not present.

Taken together, our findings demonstrate that sensory symptoms were present across individuals with ADNP syndrome regardless of age, sex, cognition, adaptive skills, and importantly, irrespective of ASD diagnosis. Sensory symptoms, particularly seeking, appear to span the range of individuals with ADNP syndrome and can be quantified using existing standardized instruments, such as the SAND which appears to be the most robust assessment, independent of functioning level or age. Sensory symptoms represent a novel target for treatment in ADNP clinical trials, and clinically, can inform treatment recommendations based on an individual’s unique sensory preferences.

## Figures and Tables

**Table 1:** Most commonly endorsed items on the Sensory Assessment for Neurodevelopmental Disorders (SAND) Interview.

Item	Domain	% Endorsed
*Does your child appear fascinated with certain textures (e.g., the feel of certain objects, water, a person’s skin)?*	Tactile Seeking	95.24%
*Does your child enjoy seeking pressure or bump or crush into objects (e.g., walls, furniture) or people?*	Tactile Seeking	85.71%
*Does your child use objects or his/her voice to create sounds outside of the context of functional play (e.g., banging toys together, repetitive sounds)?*	Auditory Seeking	85.71%
*Does your child notice hot or cold temperatures (e.g., hot bath, ice) and pain (e.g., getting a shot, hurting self)?* (reverse coded)	Tactile Hyporeactivity	80.95%

## Data Availability

The majority of the dataset used during the current study is included in this published article and Supplementary File. The remainder of the dataset is available from the corresponding author on reasonable request and may require ethics review.

## Supplementary Materials

The following are available online at https://www.mdpi.com/2073-4425/12/3/351/s1, Table S1: Genetic variants in the cohort, Table S2: Vision and hearing abnormalities in the cohort.
